# Comprehensive analysis of a-and b-thalassemia genotypes and hematologic phenotypes

**DOI:** 10.5937/jomb0-51740

**Published:** 2025-01-24

**Authors:** Wang Heng, Huang Hai, Chen Yaping, Dan Xie, An Bangquan, Huang Shengwen

**Affiliations:** 1 Guizhou Medical University, School of Clinical Laboratory Science, Guiyang, China; 2 Guiyang Public Health Clinical Center, Guiyang, China; 3 The Second People's Hospital of Guiyang, Department of Clinical Laboratory, Guiyang, China; 4 Guizhou University, Medical College, Guiyang, China; 5 Guizhou Provincial People's Hospital, Department of Blood Transfusion,Guiyang, China; 6 Guizhou Provincial People's Hospital, Department of Medical Genetics, Guiyang, China

**Keywords:** thalassemia, genotypes, phenotypes, hemoglobin, b-globin, talasemija, genotipovi, fenotipovi, hemoglobin, b-globin

## Abstract

**Background:**

Guizhou Province is an area with high incidence of thalassemia. However, there are few large-sample studies on the correlation between genotypes and phenotypes in Guizhou Province. In this study, the phenotypes and genotypes of 1174 patients with thalassemia in Guizhou Province were collected, and the relationship between different genotypes and phenotypes was analyzed, providing a more accurate basis for genetic counseling, prevention and control of thalassemia.

**Methods:**

A total of 1174 patients with thalassemia were collected in Guizhou Provincial People's Hospital from October 2020 to December 2021 by PCR-reverse dot blot (RDB) hybridization assay, and their red blood cell (RBC), hemoglobin (Hb), mean erythrocyte volume (MCV), mean corpuscular hemoglobin (MCH), mean corpuscular hemoglobin concentration (MCHC), red blood cell distribution width (RDW), hemoglobin (HbA), hemoglobin A2 (HbA2), and fetal hemoglobin (HbF) data were collected. The relationship between different genotypes and phenotypes was analyzed.

**Results:**

Among 1174 cases of thalassemia or carriers, there were 617 cases of a-thalassemia, 512 cases of b-thalassemia, 45 cases of coinheritance of aand b-tha-lassemia. The severity of anemia between a-thalassemia was positively correlated with the decrease of non-functional copy number of a-globin gene. The degree of anemia in non-deletion a-thalassemia was greater than that in deletion a-thalassemia. In b-thalassemia, b0 gene mutation did not produce b-globin, and b+ mutation expressed some bglobin, but it was lower than normal level. b0/b0 had no bglobin production, and long-term blood transfusion was required to maintain life. Compared with a-thalassemia, the degree of anemia in b-thalassemia whose clinical type was same as a-thalassemia was more serious. The anemia degree of coinheritance of aand b-thalassemia was less than that of simple a-thalassemia or b-thalassemia.

**Conclusions:**

The clinical phenotype of thalassemia is influenced by molecular mechanism, and the two kinds of thalassemia can interact with each other. The clinical severity is positively correlated with the imbalance of a peptide chain and b peptide chain. A comprehensive understanding of the hematologic phenotype differences between different genotypes and subtypes of thalassemia can provide more accurate data for genetic counseling of thalassemia.

## Abstract (sr)

**Uvod:**

Provincija Guizhou je oblast sa visokom incidencom talasemije. Međutim, postoji nekoliko studija velikih uzoraka o korelaciji između genotipova i fenotipova u provinciji Guizhou. U ovoj studiji prikupljeni su fenotipovi i genotipovi 1174 pacijenata sa talasemijom u provinciji Guidžou i analiziran je odnos između različitih genotipova i fenotipova, dajuči tačniju osnovu za genetsko savetovanje, prevenciju i kontrolu talasemije.

**Metode:**

Ukupno 1174 pacijenata sa talasemijom prikupljeno je u Narodnoj bolnici provincije Guidžou od oktobra 2020. do decembra 2021. pomoču PCR-reverse dot blot (RDB) hibridizacionog testa i njihovih crvenih krvnih zrnaca (RBC), hemoglobina (Hb), srednje vrednosti Prikupljeni su podaci zapremine (MCV), srednjeg korpuskularnog hemoglobina (MCH), srednje koncentracije korpuskularnog hemoglobina (MCHC), širine distribucije crvenih krvnih zrnaca (RDV), hemoglobina (HbA), hemoglobina A2 (HbA2) i fetalnog hemoglobina (HbF). Analiziran je odnos između različitih genotipova i fenotipova.

**Rezultati:**

Među 1174 slučaja talasemije ili nosilaca, bilo je 617 slučajeva a-talasemije, 512 slučajeva P-talasemije, 45 slučajeva konasleđivanja ai b-talasemije. Ozbiljnost anemije između a-talasemije bila je u pozitivnoj korelaciji sa smaStepen anemije kod nedelecione a-talasemije bio je veći nego kod delecione a-talasemije. Kod P-talasemije, mutacija P0 gena nije proizvela b-globin, a P+ mutacija je eksprimirala nešto P-globina, ali je bila niža od normalnog nivoa. P°/P° nije imao proizvodnju P-globina, a dugotrajna transfuzija krvi je bila potrebna da bi se održao život. U poređenju sa atalasemijom, stepen anemije kod P-talasemije čiji je klinički tip bio isti kao a-talasemija bio je ozbiljniji. Stepen konasleđivanja ai P-talasemije anemije bio je manji nego kod jednostavne a-talasemije ili P-talasemije.

**Zaključak:**

Klinički fenotip talasemije je pod uticajem molekularnog mehanizma, a dve vrste talasemije mogu da interaguju jedna sa drugom. Klinička težina je u pozitivnoj korelaciji sa neravnotežom peptidnog lanca i P peptidnog lanca. Sveobuhvatno razumevanj'e hematoloških fenotipskih razlika između različitih genotipova i podtipova talasemije može pružiti tačnije podatke za genetsko savetovanje talasemije.

## Introduction

Thalassemia, also known as marine anemia, is one of the most common monogenic genetic diseases in humans [1]. According to statistics, thalassemia was widespread in tropical and subtropical areas where malaria was prevalent [2]. The vast areas south of the Yangtze River in China are high incidence areas, especially Guangdong, Guangxi and Hainan, and Sichuan, Guizhou and Yunnan are also serious areas. Thalassemia has become a serious public health problem. The disease is a chronic hemolytic anemia caused by partial or complete inhibition of globin peptide chain synthesis, resulting in an imbalance in the number of α and non-α globin peptide chains, and the deposition of excess globin peptide chains on the erythrocyte membrane, which increases the fragility of red blood cell (RBC) and shortens their lifespan. The most common clinical cases are α-thalassemia and β-thalassemia. The α-globin gene cluster is located in the p13.3 region of chromosome 16 and mainly contains two functional regions, α1 and α2. The α-mutations can be divided into deletion and non-deletion types, with deletion type accounting for the majority. The deletion type mainly affects the α1 gene, and the non-deletion type mainly affects the α2 gene. The common deletion types in China are -α^3.7^ (right deletion type), -α^4.2^ (left deletion type) and — sea (Southeast Asian deletion type), and the common non-deletion mutation types are α ^CS^-α and α ^QS^-α. The β-globin gene is located in the p15.5 region of chromosome 11. In adulthood, it is expressed as β (HBB). The genetic variation of β-thalassemia can be divided into point mutation, small fragment deletion and large fragment deletion, most of which are point mutation and small fragment deletion. According to the genetic effect, it can be divided into β^++^ mutations, and the synthesis rate of β-globin peptide chain is slightly lower than normal. In the β^+^ mutation, bglobinpeptide chain synthesis rate decreased. In the β^0^ mutation, no β-globin peptide chain can be produced. According to the different genotype, the degree of influence on globin peptide chain expression is different, and the clinical phenotype is different. Moreover, different mutation types combined to form new and different types of thalassemia, the clinical phenotypes have great differences. Moreover, coinheritance of α- and β-thalassemia also affected the clinical phenotype. This clinically significant heterogeneity is directly related to the intracellular imbalance between α- and β-globin chains [3]. In this study, patients with α-thalassemia, β-thalassemia and coinheritance of α- and β-thalassemia diagnosed by genotype in Guizhou Provincial People’s Hospital were collected. The relationship between different genotypes and phenotypes was analyzed to provide a more accurate basis for genetic counseling and prevention of thalassemia.

## Materials and methods

### Study subjects

**Figure 1:**
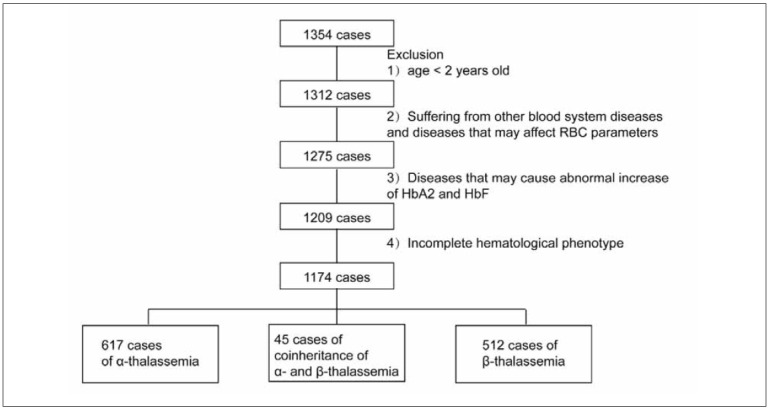
Flowchart of exclusion for 1174 cases.

A total of 1174 patients or carriers of thalassemia were diagnosed by PCR reverse dot hybridization (RCR-RDB) in Guizhou Provincial People’s Hospital from October 2020 to December 2021, including individuals from various regions, ranging in age from newborns to 86 years old, with an average age of 27.46±15.59 years old, and 513 men and 772 women. Figure 1 Exclusion: 1) age < 2 years old; 2) Suffering from other blood system diseases and diseases that may affect RBC parameters; 3) Diseases that may cause abnormal increase of HbA2 and fetal hemoglobin (HbF); 4) Incompletehematological phenotype. This study was approved by the Ethics Committee of Guizhou Provincial People’s Hospital (approval document No. 2020-05), and all subjects or their legal guardians signed informed consent.

### Genomic DNA extraction

Genomic DNA was extracted from blood samples by magnetic beadhop method of NP968 nucleic acid extraction system (Xi’an Tianlong Science and Technology, Xi’an, China). The concentration and purity of extracted DNA were assessed using a NanoDrop spectrophotometer (Thermo Fisher Technologies USA). The ratio of absorbance value of extracted DNA at OD260 nm/280 nm was between 1.5–2.5, and the concentration was 20–40 ng/mL. The extracted DNA was stored at -20°C.

### α- and β-thalassemia detected by PCR-RDB

A 5 μL single extracted DNA sample was used for PCR amplification. The amplified products weretested using the α/β-thalassemia gene detection kit (Hybriobio Co., LTD., Guangzhou, China) in theautomatic nucleic acid molecule hybridization instrument (HBHM-3000S, Hybriobio Co., LTD., Guangzhou, China). In the Chinese population, the method was used to detect 3 common α-thalassemia deletion types (—^SEA,^ -α ^3.7^, -α ^4.2^), 3 α-thalassemia non-deletion types (Hb CS (Constant Spring), Hb QS (Quong Sze), Hb WS (Westmead)), and 19 known β-thalassemia mutations at 17 sites, CD41-42(-CTTT), CD43(G > T), IVS-II-654 (C > T), CD17(A > T), CD14-15(+G), -28(A > G), -29(A > G), CD71-72(+A), βE(G > A), IVS-II-1(G > A/T), CD27-28(+C), IVS-II-5(G > C), CAP(A > C/-AAAC), Int(T > G), CD31(-C), -30(T > C), and -32(C > A), respectively. Testing was performed according to the manufacturer’s protocol.

### Analysis for erythrocyte parameters

RBC parameters were analyzed using an automatic blood cell analyzer (Sysmex XN-100-4, Japan), and automatic electrophoresis analysis system (Hydrasys LC; Sebia Electrophoresis, Evry, France) was used for hemoglobin (Hb) analysis. For adults, the RBC reference range was 4.0–5.50×10^12^ /L, the reference range of Hb was 120–160 g/L, the reference range of mean erythrocyte volume (MCV) was 80–100 fL, the reference range of mean corpuscular hemoglobin (MCH) was 27–34 pg, and the reference range of mean corpuscular hemoglobin concentration (MCHC) was 320–360 g/L. The reference range of red blood cell distribution width (RDW) was 35.0–56.0 fL.

### Statistical analysis

GraphPad Prism 8 software system was used for statistical analysis of the data. Normal distribution data were expressed as mean ± standard deviation, and t-test was used to compare the difference between the two groups. One-way ANOVA test was used to compare multiple groups. Skewness data were expressed as median and interquartile interval (M, p), and differences between groups were compared using non-parametric Mann-Whitney test. P < 0.05 indicated that the difference was statistically significant.

## Results

### Distribution of thalassemia genotypes

From October 2020 to December 2021, 1174 patients with thalassemia were diagnosed in Guizhou Provincial People’s Hospital, including 617 cases of α -thalassemia, 512 cases of β-thalassemia, and 45 cases of coinheritance of α - and β-thalassemia (Table 1).

**Table 1:** Distribution of thalassemia genotypes in 1174 cases.

Groups	Types	Genotypes	Number of cases (n)	Proportion (%)
Static α-thalassemia	Deletion	-α^3.7^/αα	72	11.67
		-α^4.2^/αα	40	6.48
	Non-deletion type 1	α^CS^α/αα	57	9.24
		α^QS^α/αα	7	1.13
	Non-deletion type 2	α^WS^α/αα	13	2.11
Mild α-thalassemia	Deletion	—^SEA^/aa	365	59.16
		-α^3.7^/-α^3.7^	8	1.30
		-α^4.2^/-α^4.2^	1	0.16
		-α^3.7^/-α^4.2^	1	0.16
	Non-deletion	-α^3.7^/α^CS^α	3	0.49
		α^CS^α/α^QS^α	3	0.49
		α^CS^α/α^CS^α	2	0.32
Intermediate α-thalassemia	Deletion	—^SEA^/-α^3.7^	23	3.73
		—^SEA^/-α^4.2^	5	0.81
	Non-deletion	—^SEA^/αCSα	16	2.59
		—^SEA^/α^WS^α	1	0.16
Mild β-thalassemia	β^+^/β^N^	β^IVS^-II-^654^/β^N^	72	14.06
		β^E^/β^N^	16	3.13
		β^-28^/β^N^	5	0.98
		β^-29^/β^N^	3	0.59
		β^Cap^/β^N^	2	0.39
	β^0^/β^N^	β^CD17^/β^N^	211	41.21
		β^CD41-42^/β^N^	166	32.42
		β^CD71-72^/β^N^	7	1.37
		β^CD43^/β^N^	6	1.17
		β^CD27-28^/β^N^	2	0.39
		β^CD14-15^/β^N^	1	0.20
Intermediate β-thalassemia	β^+^/β^+^	β^-28^/β^-28^	2	0.39
Severe β-thalassemia	β^+^/β^0^	β^CD17^/β^E^	5	0.98
		β^CD41-42^/β^-28^	2	0.39
		β^-29^/β^CD17^	1	0.20
		β^-29^/β^CD27-28^	1	0.20
		β^CD41-42^/β^E^	2	0.39
	β^0^/β^0^	β^CD17^/β^CD17^	3	0.59
		β^CD41-42^/β^CD17^	3	0.59
		β^CD41-42^/β^CD41-42^	1	0.20
		β^CD41-42^/β^CD27-28^	1	0.20
Coinheritance of static α-thalassemia<br>and mild β-thalassemia	-α^3.7^/αα; β^CD17^/β^N^	12	26.67	
	-α^4.2^/αα; β^CD41-42^/β^N^	6	13.33	
	α^CS^/αα; β^CD17^/β^N^	4	8.89	
	-α^3.7^/αα; β^CD41-42^/β^N^	3	6.67	
	-α^3.7^/αα; β^IVS-II-654^/β^N^	2	4.44	
	-α^4.2^/αα; β^CD17^/β^N^	3	6.67	
	α^QS^/αα; β^CD41-42^/β^N^	1	2.22	
	-α^3.7^/αα; β^E^/β^N^	1	2.22	
Coinheritance of mild α-thalassemia<br>and mild β-thalassemia	—^SEA^/αα; β^CD17^/β^N^	3	6.67	
	—^SEA^/αα; β^CD41-42^/β^N^	4	8.89	
	—^SEA^/αα; β^IVS-II-654^/β^N^	1	2.22	
	-α^3.7^/-α^3.7^; β^CD17^/β^N^	1	2.22	
Coinheritance of intermediate<br>α-thalassemia and mild β-thalassemia	—^SEA^/α^CS^α; β^CD17^/β^N^	2	4.44	
	—^SEA^α^CS^α; β^CD41-42^/β^N ^	1	2.22	
Coinheritance of intermediate<br>α-thalassemia and severe β-thalassemia	—^SEA^/α^CS^α; β^CD17^/β^CD17^	1	2.22	
**Total**			**1174**	**100**

There were 189 cases of static α-thalassemia, 383 cases of mild α-thalassemia, and 45 cases of intermediate α-thalassemia in α-thalassemia. There were 112 cases of static α-thalassemia (deletion type), 64 cases of static α-thalassemia (non-deletion type 1) (CS and QS), and 13 cases of static α-thalassemia (non-deletion type 2) (WS). There were 375 cases of mild α-thalassemia (deletion type) and 8 cases of mild α-thalassemia (non-deletion type). There were 28 cases of intermediate α-thalassemia (deletion type) and 17 cases of intermediate α-thalassemia (non-deletion type). Among all α-thalassemia genotypes, —SEA/αα (Southeast Asian deletion type) was the most common (365 cases, 59.16%), followed by -α^3.7^/αα (right side deletion) (72 cases, 11.67%), and the third was α^CSα^/αα (57 cases, 9.24%) (Table 1).

In the β-thalassemia, there were 491 cases of mild β-thalassemia, 2 cases of intermediate β-thalassemia (β^+^/β^+^) and 21 cases of severe β-thalassemia (β^+^/b^0^) (b^0^/β^0^). There were 98 cases ofmild β-thalassemia (β^+^/β^N^) and 393 cases of mild β-thalassemia (β^0^/β^N^). There were 11 cases of severe β-thalassemia (β^+^/β^0^) and 8 cases of severe β-thalassemia (β^0^/β^0^). The top three gene frequencies were β^CD17^/β^N^ (211 cases, 41.21%), β^CD41-42^/β^N^ (166 cases, 32.42%) and β ^IVS-II-654^ /β^N^ (72 cases, 14.06%) (Table 1).

In the coinheritance of α- and β-thalassemia, the most common genotype frequency was -α^3.7^/αα; β^CD17^/β^N^ (12 cases, 26.67%) (Table 1).

### Analysis for hematologic phenotypes of different genotypes of thalassemia

According to different thalassemia genotypes (Table 2), there was statistical significance in different clinical phenotypic indexes including RBC, Hb, MCV, MCH, MCHC, RDW, adult hemoglobin (HbA), and HbA2 among each group (P < 0.05).

**Table 2:** Hematologic phenotypes of different genotypes of thalassemia.

Groups	Number<br>of cases<br>(n)	RBC<br>(10^9^/L)	Hb (g/L)	MCV (Fl)	MCH (pg)	MCHC (g/L)	RDW (%)	HbA (%)	HbA2 (%)	HbF<br>(M,Q)
Static a-thalassemia<br>(deletion)	112	4.66±0.53	127.6±16.4	83.31±4.08	27.55±1.72	329.5±11.7	40.97±2.62	97.42±0.69	2.40±0.29	0.8(29.1)
Static a-thalassemia<br>(Non-deletion type 1)	64	4.73±0.67	124.4±20.0	80.37±5.84	26.32±2.53	327.3±15.6	40.37±4.60	97.00±0.87	2.20±0.23	0.7(4.2)
Static a-thalassemia<br>(Non-deletion type 2)	13	4.63±0.53	128.8±15.6	83.73±8.21	27.98±3.03	334.7±12.1	40.56±2.85	97.15±0.27	2.72±0.15	0.3(0.5)
Mild α-thalassemia<br>(deletion)	375	5.20±0.86	121.5±18.1	73.45±8.67	23.73±3.66	319.8±19.3	38.77±5.51	96.89±5.20	2.38±0.26	0.7(36.2)
Mild α-thalassemia<br>(non-deletion type)	8	4.25±0.90	102.5±19.8	78.24±6.09	24.25±2.43	309.8±13.1	43.38±4.23	95.34±1.84	1.73±0.28	0.5(2.4)
Mild β-thalassemia(β^+^/β^N^)	98	5.26±0.96	112.8±19.4	68.11±9.06	21.86±4.09	319.9±18.9	38.42±9.27	88.59±9.90	5.00±2.45	1.9(14.3)
Mild β-thalassemia<br>(β^0^/β^N^)	393	5.36±1.06	108.5±19.4	64.16±7.26	20.38±2.32	315.8±19.4	37.77±18.18	92.41±5.49	5.41±0.52	1.3(12.3)
Intermediate<br>α-thalassemia (deletion)	28	4.99±0.97	91.5±16.4	62.37±6.31	18.47±1.57	297.2±15.8	48.55±7.17	89.89±13.48	1.10±0.27	1.1(1.2)
Intermediate α-thalassemia<br>(non-deletion type 1)	16	3.91±0.83	78.8±11.9	75.84±10.57	20.59±2.54	272.1±17.5	63.24±12.77	83.96±7.03	1.04±0.45	1.1(1.7)
Intermediate α-thalassemia<br>(non-deletion type 2)	1	6.46	134.0	64.7	20.7	320	14.7	97.8	2.2	
Intermediate β-thalassemia<br>(β^+^/β^+^)	2	4.62	92.0	65.08	20.00	307.3		52.90	6.80	40.3
Severe β-thalassemia<br>(β^+^/β^0^)	11	3.21±0.66	71.6±16.4	73.26±8.74	22.42±3.16	305.5±12.1	59.90±21.47	24.35±16.79	3.04±0.76	69.3(38.5)
Severe β-thalassemia<br>(β^0^/β^0^)	8	3.23±1.15	77.5±28.2	75.44±7.45	23.99±3.31	316.8±15.3	57.46±11.88	15.74±33.98	2.97±0.56	96.5(91.4)

Hb from high to low was static α-thalassemia (non-deletion type 2) (αWSα), static α-thalassemia (deletion type), static α-thalassemia (non-deletion type 1) (αCs-α, αQs-α), mild α-thalassemia (deletion type), mild β-thalassemia (β^+^/β^N^), mild β-thalassemia (β^0^/β^N^), Mild alpha-thalassemia (non-deletion type) (excluding WS), intermediate β-thalassemia (β^+^/β^+^), intermediate α-thalassemia (deletion type), intermediate α-thalassemia (non-deletion type), severe β-thalassemia (β^0^/β^0^), and severe β-thalassemia (β^+^/β^0^) (Table 2). There was no significant difference between static α-thalassemia (deletion type) (127.6±16.4) and static α-thalassemia (non-deletion type 1) (124.4±20.0), with t=1.018 and P=0.3103 > 0.05. There was a significant difference between mild α-thalassemia (deletion type) (121.5±18.1) and mild α-thalassemia (non-deletion type) (102.5±19.8), with t value of 2.929 and P=0.0036 < 0.01. There was a difference between α-thalassemia intermedia (deletion type) (91.5±16.4) and α-thalassemia intermedia (non-deletion type) (78.8±11.9), t=2.535, P=0.0154 < 0.05. There was no significant difference between β-thalassemia minor (β^+^/β^N^) (112.8±19.4) and β-thalassemia minor (β^0^/β^N^) (108.5±19.4), with t=1.911, P=0.0566 > 0.05. There were only 2 cases of intermediate β-thalassemia (β^+^/β^+^), and the mean number was 92.0, which was higher than that of severe β-thalassemia (β^+^/β^0^, 71.6) and severe β-thalassemia (β^0^/β^0^, 77.5). There was no significant difference between severe β-thalassemia (β^+^/β^0^) (71.6±16.4) and severe β-thalassemia (β^0^/β^0^) (77.5±28.2), with t value of 0.5811 and P=0.5688 > 0.05. There was a significant difference between mild α-thalassemia (121.1±18.4) and mild β-thalassemia (109.9±18.7), with t=8.653, P< 0.001. There was a significant difference between intermediate α-thalassemia (88.8±17.8) and intermediate or severe β-thalassemia (75.8±21.2), with t = 2.595, P = 0.0117.

**Figure 2:**
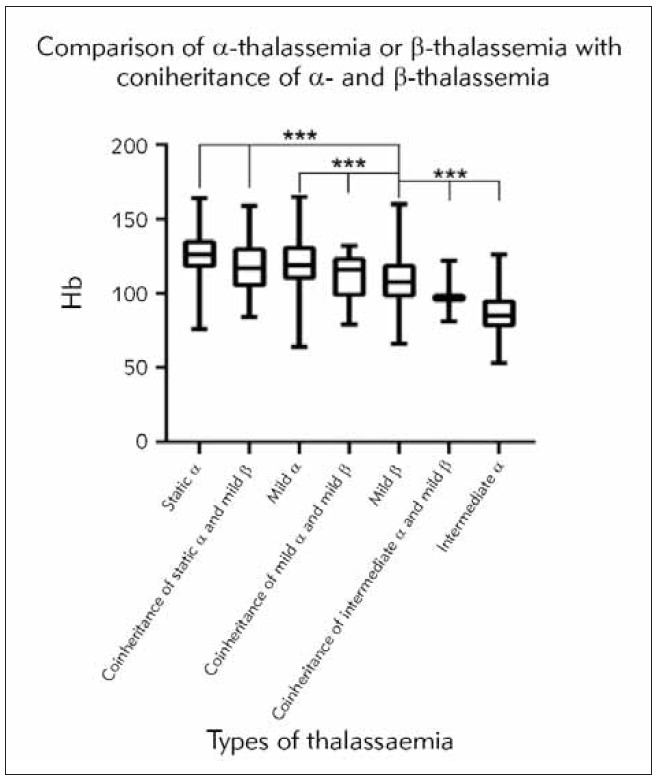
Comparison of Hb in α-thalassemia group, β-thalassemia group and α- and β-thalassemia group. *** represent *p*<0.001

There were significant differences in Hb among the three groups of static α-thalassemia (125.6± 19.2), coinheritance of static α-thalassemia and mild β-thalassemia (120.8±18.3), and mild β-thalassemia (108.7±19.9) (F = 52.13, P < 0.0001). There were significant differences in Hb among the three groups of mild α-thalassemia (121.1±18.4), coinheritance of mild α-thalassemia and mild β-thalassemia (114.5±18.6), and mild β-thalassemia (108.7±19.9) (F=44.58, P<0.0001). There were significant differences in Hb among the three groups of intermediate α-thalassemia (87.49±16.22), coinheritance of intermediate α-thalassemia and mild β-thalassemia (100.0±20.7), and mild β-thalassemia (108.7±19.9) (F =23.71, P < 0.0001) (Figure 2).

One case was a 16-year-old young female with the genotype —^SEA^/α^CS^α; β^CD17^/β^CD17^, with coinheritance of intermediate α-thalassemia (non-deletion type) and severe β-thalassemia. Hematological phenotype Hb was 52 g/L, showing severe small cell hypochromic anemia, and HbF up to 91.6% (Table 3).

**Table 3:** Hematologic phenotypes of coinheritance of α- and β-thalassemia. Comparation of HBA2 in static a and mild a group, intemediate a group, severe b group and mild b group. **** represent p 0.0001

Groups	Number<br>of cases<br>(n)	RBC<br>(10^9^/L)	Hb<br>(g/L)	MCV<br>(Fl)	MCH<br>(pg)	MCHC<br>(g/L)	RDW<br>(%)	HbA<br>(%)	HbA2<br>(%)	HbF<br>(%)
Coinheritance of<br>static α-thalassemia<br>and mild<br>β-thalassemia	32	5.67±0.92	120.8±18.3	66.23±4.95	21.41±1.93	322.5±13.6	36.79±4.03	93.28±4.35	5.37±0.45	1.30<br>(20.6)
Coinheritance of<br>mild α-thalassemia<br>and mild<br>β-thalassemia	9	5.23±0.22	114.5±18.6	68.80±7.39	21.92±3.43	316.9±19.4	40.16±11.53	94.78±1.20	4.83±1.08	0.35<br>(1.0)
Coinheritance of<br>intermediate<br>α-thalassemia and<br>mild β-thalassemia	3	6.58±1.53	100.0±20.7	52.43±3.31	15.27±0.60	295.9±10.6	32.85±1.49	94.57±0.67	3.13±0.25	0<br>(1.4)
Coinheritance<br>of intermediate<br>α-thalassemia<br>and severe<br>β-thalassemia	1	2.56	52.0	67.19	20.30	302.3			6.40	91.6

Only three groups had a mean erythrocyte count below 4×10^9^/L: intermediate α-thalassemia (non-deletion type), severe β-thalassemia (β^0^/β^0^), and intermediate or severe β-thalassemia (β^+^/β^0^). With Hb decline, there were no decreases in RBCs in static α-thalassemia (non-deletion type 2) (α^WS^-α), static α-thalassemia (deletion type), static α-thalassemia (non-deletion type 1) (α^CS^-α, α^QS^-α), mild α-thalassemia (deletion type), mild β-thalassemia β^+^/β^N^), and mild β-thalassemia (β^0^/β^N^), but the trend was gradually increasing. With the decrease of Hb, MCV and MCH showed a downward trend for all types of thalassemia, except for mild α-thalassemia (non-deletion type), intermediate α-thalassemia (non-deletion type), severe β-thalassemia (β^0^/β^0^) and severe β-thalassemia (β^+^/β^0^). In the static α-thalassemia, MCV of non-deletion type and deletion type was 83.73±8.21 and 83.31±4.08, P>0.05, and MCH of non-deletion type and deletion type was 27.98±3.03 and 27.55±1.72, P>0.05. In the mild α-thalassemia, MCV of non-deletion type and deletion type was 78.24±6.09 and 73.45±8.67, P>0.05, and MCH of non-deletion type and deletion type was 24.25±2.43 and 23.73±3.66, P>0.05. In the intermediate α-thalassemia, MCV of non-deletion type and deletion type was 75.84±10.57 and 62.37±6.307, P<0.0001, and MCH of non-deletion type and deletion type was 27.98±3.03 and 27.55±1.72, P<0.01. In the mild β-thalassemia, MCV of β^+^/β and β^0^/β was 68.11±9.059 and 64.16±7.259, P<0.0001; MCH of β^+^/β and β^0^/β was 21.86±4.087 and 20.38±2.319, P<0.0001; HBA2 of β^+^/β and β^0^/β was 4.999±2.445 and 5.406±0.5226, P<0.01. With the decrease of Hb, MCHC showed a gradually decreasing trend, except for intermediate β-thalassemia (β^+^/β^+^), severe β-thalassemia (β^0^/β^0^) and severe β-thalassemia (β^+^/β^0^). There were three groups with RDW > 56%: intermediate α-thalassemia (non-deletion type), severe β-thalassemia (β^0^/β^0^), and severe β-thalassemia β^+^/β^0^). There were static α-thalassemia (deletion type), static α-thalassemia (non-deletion type 1), mild α-thalassemia (deletion type), mild α-thalassemia (non-deletion type), intermediate α-thalassemia (deletion type), intermediate α-thalassemia (deletion type), and intermediate α-thalassemia (non-deletion type) with HbA2 mean less than 2.5. There were mild β-thalassemia (β^+^/β^N^), mild β-thalassemia (β^0^/β^N^) and intermediate β-thalassemia β^+^/β^+^) with HbA2 greater than 3.5. HbA2 of mild α-thalassemia and mild β-thalassemia was 2.363±0.2784 and 5.312±1.187, P<0.0001. HbA2 of intermediate α-thalassemia and intermediate or severe β-thalassemia was 1.094±0.3438 and 3.279±1.190, P<0.0001. HbF of mild α-thalassemia and mild β-thalassemia was 0.65 (36.2) and 1.4 (14.3), P<0.0001. HbF of intermediate α-thalassemia and intermediate or severe β-thalassemia was 0.85 (1.2) and 73.75 (91.4), P < 0.0001.

### Comparison of hemoglobin components in different genotypes of thalassemia

**Figure 3:**
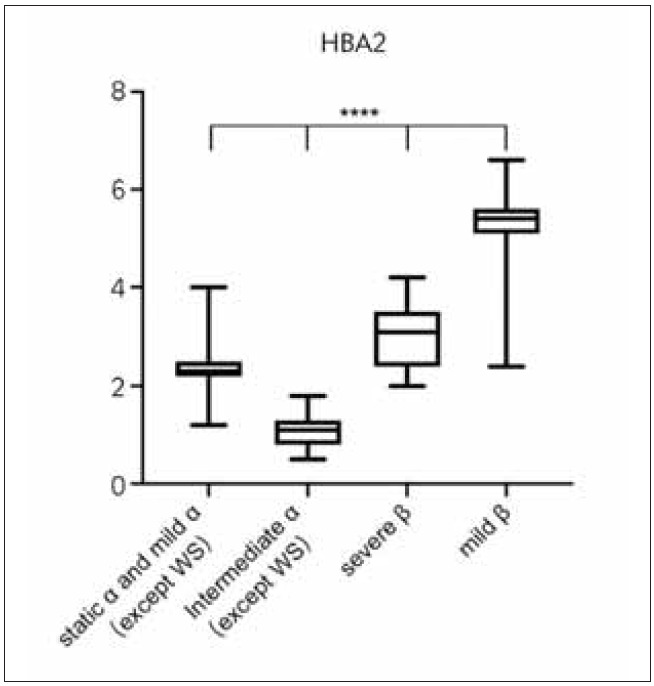
Comparation of HBA2 in static α and mild α group, intemediate α group, severe β group and mild β group. **** represent *p*<0.0001

In all thalassemia genotypes (excluding complex β-thalassemia), there were significant differences in HbA2 content. After the elimination of static α-thalassemia (non-deletion type 2) as normal group and intermediate β-thalassemia with fewer cases, the remaining genotypes could be divided into 4 groups according to the content of HbA2: Static and mild α-thalassemia (2.35±0.28), mild β-thalassemia (5.26±0.66), intermediate α-thalassemia (1.09±0.34) and severe β-thalassemia (3.01±0.65). According to multi-group variance analysis, there were statistically significant differences in HbA2 content among the four groups (P < 0.0001) (Figure 3).

In addition, the HbA2 content of the two types of mild β-thalassemia (β^+^/β^N^ and β^0^/β^N^) was significantly different (t=8.88, P < 0.0001). HbF content was significantly increased in intermediate and severe β-thalassemia. The Hb composition of static and coinheritance of mild α-thalassemia and mild β-thalassemia was similar to mild β-thalassemia.

## Discussion

In this study, 1174 patients with α-thalassemia, β-thalassemia and coinheritance of α- and β-thalassemia detected by PCR were collected from Guizhou Provincial People’s Hospital from October 2020 to December 2021. Our results only targeted at common mutation types, excluding rare mutation types. This is a large sample survey, obtained the relevant data of Guizhou thalassemia. Among the thalassemia detected in our hospital, the most common types of a-thalassemia were Southeast Asian deletion —^SEA^/αα (256,38.38%), right deletion — α^3.7^/αα(216,32.38%), α^CS^-α /αα (60,9.00%), and left deletion — α^4.2^/αα (40,6.00%). This was different from the genotype sequencing of α-thalassemia in Guizhou reported in the data from November to June 2008 [4]. The frequency of α^CS^-α /aa gene increased significantly, surpassing that of L-deletion - α^4.2^/αα, which was similar to the gene frequency distribution in Yunnan Province at that time. The reason may be that with the migration of people between the two provinces, the frequency of α^CS^-α / α-α gene was increased in our province. As this result may lead to an increase in the frequency of the intermediate α-thalassemia (non-deletion) gene, which is the type of thalassemia with a more severe clinical phenotype [5], it will become more important to count areas with high frequency of the α^CS^-α /aa gene, and to do a good job in the screening and prenatal counseling of thalassemia genes in these areas. The most common genotypes of β-thalassemia were β^CD17^/β^N^ (236, 41.92%), β^CD41-42^/β^N^ (180, 31.97%) and β^IVS-II-654^/β^N^ (77, 13.68%). This is consistent with our report in 2015, and consistent with the frequency of β-thalassemia gene in neighboring provinces Sichuan and Chongqing [6].

There are many studies on the differences between α-thalassemia and β-thalassemia genotypes and hematologic phenotypes, but few studies have comprehensively analyzed the genotypes of the two types of thalassemia. Our results show that there are indeed differences in hematologic phenotypes between different types of thalassemia and different genotypes. Previously, α-thalassemia was divided into static type, mild (or standard) type, HbH disease (or intermediate type), Hb Bart’s fetal edema syndrome (or severe type) [7], and β-thalassemia was divided into static type, mild type, intermediate type, and severe type [8]. Children with severe α-thalassemia were not included in this study because they did not survive. Static β-thalassemia gene is mainly common in Mediterranean population [9], which is not common in China and was not included in this study. It was found that the hematologic phenotypes of thalassemia, especially the intermediate thalassemia, varied greatly from mild to severe anemia. The complex genotype-phenotype relationship cannot be well explained by the above classification alone. Therefore, α-thalassemia and b-thalassemia were classified into more detailed types. The α-thalassemia was classified as static α-thalassemia (deletion type) - α/αα, static α-thalassemia (non-deletion type 1) α^T^α/αα (except α^W^S-α/αα), static α-thalassemia (non-deletion type 2) α^WS^α/αα, mild α-thalassemia (deletion type) —/αα or -α/αα, mild α-thalassemia (non-deletion type) -α/αα^T^ or α^T^α/α^T^α, intermediate α-thalassemia (deletion type) —/-α, intermediate α-thalassemia (non-deletion type) —/α^T^α. The β-thalassemia was classified as mild β-thalassemia (β^+^/β^N^), mild β-thalassemia (β^0^/β^N^), intermediate bthalassemia (β^+^/β^+^), severe β-thalassemia (β^+^/β^0^), and severe β-thalassemia (β^0^/β^0^).

Our results showed that the highest hemoglobin was static α-thalassemia (non-deletion type 2) (α^WS^-α). There was one case of intermediate α-thalassemia with genotype of —^SEA^/α^WS^-α, which also showed no anemic characteristics, with a hemoglobin of 134 g/L. This suggested that α^WS^ was a recessive a mutation. This is in line with relevant research results [10], Hb-Westmead is a silent non-deletion α+ mutation, and the degree of α-globin chain deletion is less than other non-deletion α-mutations or deletion α-mutations. With the increase of α-globin gene deletion, apeptide chain synthesis decreased, and the degree of anemia gradually increased, indicating that the severity of anemia between α-thalassemia and the decrease of non-functional copy number of α-globin gene was positively correlated [11]. Our study found that in α-thalassemia, with the loss of α-globin gene, hemoglobin gradually decreased, while MCV, MCH, and MCHC also gradually decreased, suggesting that at the same time when hemoglobin decreased, RBC hyperplasia was active, the proportion of naive RBCs increased, the volume reduced, and the average hemoglobin volume also decreased. In β-thalassemia, according to the clinical classification from mild to severe β-thalassemia, hemoglobin was gradually decreased, but MCV, MCH, and MCHC were not gradually decreased with the decrease of hemoglobin, showing irregular changes, which may be due to our intermediate β-thalassemia data were less, severe β-thalassemia patients may need long-term blood transfusion, which had an impact on the results of RBC parameters. In the comparison between α-thalassemia and β-thalassemia, Hb levels of β-thalassemia were lower than those of α-thalassemia in the same clinical classification, so it was concluded that β-thalassemia was the type of thalassemia with more severe clinical phenotype.

Except for the α^WS^-α genotype, the degree of anemia of α-thalassemia (non-deletion) is greater than that of α-thalassemia (deletion), because the non-deletion mutation of α-thalassemia mainly affects the α2 gene and the deletion mutation of α-thalassemia mainly affects the α1 gene, and the α2 gene accounts for two-thirds of the expression of the apeptide chain and the α1 gene accounts for onethird of α-peptide chain expression [3] [12] [13]. Liebhaber et al. [14] found that the expression of the α1-globin gene located on the same chromosome as the Hb-CS mutation was only half that of -α^3.7^ and -α^4.2^. MCV and MCH of α-thalassemia (non-deletion type) were higher than α-thalassemia (deletion type), especially the intermediate α-thalassemia was significantly different. It is speculated that the absolute number of α-peptide chain and the imbalance between α-peptide chain and β-peptide chain of deletion mutation reduce the production of hemoglobin, and hemoglobin is the main component of RBCs, and the reduction of hemoglobin synthesis will seriously affect the differentiation and maturation of RBCs; Non-deletion mutations, α^CS^ and α^QS^, will affect the production of stop codons at bit 142 of the coding sequence, resulting in an extended α-globin chain [15], which does not affect the combination of α-peptide chain and β-peptide chain, and is more conducive to the differentiation and maturation of RBCs. However, the bound hemoglobin variants are extremely unstable and prone to hemolysis. This explains why non-deletion mutations have more severe anemia, but higher MCV and MCH. It is also because the non-deletion type has more severe anemia, but the cells are enlarged, so the MCHC is lower. Previous study had also pointed out that the oxidation of excess β chain and a chain led to the damage of cell membrane, which led to the production of EPO and the reduction of RBCs in the non-deletion genotype of α-thalassemia [16].

It is generally believed that β^0^ completely inactivates the β gene, does not produce β-globin, and β^+^ expresses some β-globin, but it is lower than the normal level [17]. In mild β-thalassemia, the degree of anemia in β^0^/β group should be higher than that in β^+^/β group. Our results showed that the degree of anemia in β^0^/β group was greater than that in β^+^/β group, but there was no statistical difference between the two groups. However, MCV, MCH and MCHC in β^0^/β group were lower than those in β^+^/β group, and HBA2 in β^0^/β group was higher than those in β^+^/β group, which reflected the decrease of β-globin chain generation, the increase of HBA2 synthesis and the increase of α inclusion bodies in β^0^ group, resulting in increased destruction of RBCs, so the cell size was smaller and the hemoglobin in each RBC was lower. Because there is a normal β gene that can continuously express a high level of β peptide chain, the degree of anemia is often not serious, and the symptoms are not serious. Although there are only two cases of intermediate β-thalassemia (β^+^/β^+^), the degree of anemia is less than β^+^/β^0^ and β^0^/β^0^. β^0^/β^0 ^genotype is a type that does not produce β-globin genes and requires long-term blood transfusion to maintain life [18]. Our study showed that there was no significant difference in the degree of anemia between β^+^/β^0^ and β^0^/β^0^, and it was related to long-term blood transfusion of patients, which also explained the high MCV and MCH in β^+^/β^0^ and β^0^/β^0^ genotypes.

HbA2 and HbF are two important indicators for thalassemia screening, and the HbA2 reference interval is generally considered to be (2.5%-3.5%), but the test results may be affected by a variety of factors, such as gender, race, region, pregnancy status, and detection method [19]. Therefor, there is no uniform reference interval for these two indicators in different populations. The cut-off value of HbA2 also varies with different factors. Our study found that HbA2 of mild β-thalassemia was significantly higher than that in mild α-thalassemia, and the results of HbA2 had suggestive value for the prediction of α-thalassemia or β-thalassemia. For patients suspected of thalassemiabefore genetic examination, HbA2 was normal or slightly decreased, and the possibility of α-thalassemia was greater, while HbA2 was significantly increased, indicating the possibility of β-thalassemia was greater. The concentration of HbF, also known as fetal hemoglobin, gradually decreases after birth, while the concentration of HbA gradually increases. Typically, adults have only a small amount of HbF in their blood. In recent years, studies have found that reactivation of γ-globin gene (γ-gene) in adult body to express more γ-globin can bind excess α-globin to form HbF to partially compensate for the deficiency of HbA, thereby improving the anemia status of thalassemia patients, reducing complications, and reducing mortality. At present, domestic and foreign studies have found that BCL11A, HBSIL-MYB, Xmnl-HBG2 [20] and other genes are involved in the regulation of γ-globin. Further analysis of the relationship between SNP loci and HbF expression may provide guidance for clinical medication, gene therapy and individualized medicine of β-thalassemia.

In coinheritance of α- and β-thalassemia, our study found that the degree of anemia was between the two types of thalassemia. In the absence of a peptide chains due to α-thalassemia, the excess β and γ peptide chains are polymerized to form β_4_ (HbH) and γ_4_ (Hb Bart’s). Among them, HbH is the most common. HbH is a kind of non-implicit Hb, which is prone to self-degeneration in RBCs and precipitate into inclusion bodies and Heinz bodies, resulting in shortened RBC life and hemolysis [21]. The β-thalassemia gene mutation leads to reduced or no synthesis of b peptide chain, and the excess a peptide chain precipitates in mature RBCs and young RBCs to form α inclusion bodies due to its unstable properties, resulting in the destruction of RBCs and anemia [22]. In other words, subunit imbalance is the core of thalassemia pathophysiology, and the degree of imbalance is proportional to the severity of the disease. The coinheritance of α- and β-thalassemia leads to the simultaneous reduction of the unstable α peptide chain and β peptide chain, the absolute number of unstable RBCs is reduced, and the hemolysis is also reduced, so the degree of anemia is lower than that of the more severe thalassemia type [23] [24]. Our study collected a case of a 16-year-old young female with the genotype —^SEA^ / α^CS^-α; β^CD17^/β^CD17^, who is a patient with coinheritance of intermediate α-thalassemia (non-deletion type) and severe β-thalassemia. Hematological phenotype Hb was 52 g/L, showing severe small cell hypochromic anemia, HbF was up to 91.6%. This patient was the most severe coinheritance of the two kinds of thalassemia in this study, which could indicate that α and β peptide chains were significantly reduced at the same time, which will seriously affect Hb synthesis, and the clinical phenotype was still severe anemia, requiring longterm blood transfusion to maintain life. In our study, the absolute quantity of coinheritance of intermediate α-thalassemia and mild β-thalassemia is small, and the quantity should be further collected to reduce statistical errors.

Through prevention and control measures, the number of children born with thalassemia has been significantly reduced, but some challenges remain. Firstly, with the rapid development of economy, population migration is more and more frequent. Migration from thalassemia endemic areas to non-thalassemia areas poses a challenge for the prevention and control of thalassemia in non-thalassemia regions where there is no well-established prenatal diagnostic system. Secondly, common thalassemia mutations do not fully explain the phenotype. The genotypic phenotypic relationships of some thalassemia types are complex and may be related to some genetic modifications associated with globin loci. These mechanisms are not yet fully understood.

## Conclusion

In summary, the hematologic phenotypes of different α-and β-thalassemia genotypes in this studywere analyzed to comprehensively understand the hematologic phenotypic differences between different genotypes and subtypes of thalassemia, providing more accurate data for genetic counseling of thalassemia, so that genetic counseling can be refined to the clinical phenotypes of different genotypes and subtypes, and be more targeted.

## Dodatak

### Consent for publications

The author read and approved the final manuscript for publication.

### Ethics approval and consent to participate

This study was approved by the Medical Ethics Committee at Guizhou Provincial People’s Hospital (approval number 2022–05).

### Informed consent

All subjects or their legal guardians signed informed consent.

### Availability of data and material

The data that support the findings of this study are available from the corresponding author upon reasonable request.

### Authors’ contributions

Heng Wang: Conceptualization, methodology, writing original draft preparation. Hai Huang, Yaping Chen, Dan Xie: Investigation, software, statistical analysis. Bangquan An, Shengwen Huang: Reviewing and editing, funding acquisition, supervision. All authors read and approved the final manuscript.

### Funding

This work was supported by the National Natural Science Foundation of China (81960040); Guizhou Provincial People’s Hospital Foundation (GPPH-NSFC-D-2019-13; 2020XM04).

### Acknowledgements

The authors appreciate all individuals who participated in this study.

### Conflict of interest statement

All the authors declare that they have no conflict of interest in this work.
